# Consumption and physico‐chemical characteristics of smoked and smoked‐dried fish commonly produced in South Benin and contribution to recommended nutrient intakes

**DOI:** 10.1002/fsn3.1763

**Published:** 2020-07-19

**Authors:** Ogouyôm Herbert Iko Afé, Mahunan François Assogba, Dona Gildas Hippolyte Anihouvi, Ben‐Sadek Boukari, Caroline Douny, Yénoukounmè Euloge Kpoclou, Balbine Amoussou Fagla, Ahmed Igout, Jacques Mahillon, Victor Bienvenu Anihouvi, Marie‐Louise Scippo, Djidjoho J. Hounhouigan

**Affiliations:** ^1^ Laboratory of Food Analysis Department of Food Sciences Faculty of Veterinary Medicine Fundamental and Applied Research for Animals and Health (FARAH) Veterinary Public Health University of Liège Liège Belgium; ^2^ Laboratory of Food Sciences School of Nutrition and Food Sciences and Technology Faculty of Agronomic Sciences University of Abomey‐Calavi Jericho Cotonou Benin; ^3^ Laboratory of Food and Environmental Microbiology Earth and Life Institute ‐ Applied Microbiology Louvain‐la‐Neuve Belgium; ^4^ Department of Biomedical and Preclinic Sciences Faculty of Medicine University of Liège Liège Belgium

**Keywords:** EPA and DHA, macronutrient daily intake, Omega 3 and Omega 6, traditional smoking

## Abstract

The work aims to assess the consumption and the physico‐chemical characteristics of smoked fish and smoked‐dried fish commonly produced and consumed in the South Benin. The consumption data were obtained from a survey involving 250 consumers, conducted in selected production localities of the Southern part of Benin, where 36 samples of smoked fish and smoked‐dried fish were collected. The highest protein contents (dry matter) were recorded in *Cypselurus cyanopterus* (85.1 ± 2.3%) and *Sphyraena barracuda* (84.5 ± 4.2%), while the highest lipid contents were recorded in *Scomber scombrus* (39.0 ± 9.2%) and *Ethmalosa fimbriata* (22.1 ± 6.3%). Smoked and smoked‐dried fish produced in South Benin contained 0.1%–12.5% (of total fatty acids) eicosapentaenoic acid and 0.1%–33.2% docosahexaenoic acid, which are the most abundant omega 3 polyunsaturated acids in these fish products. The median consumption of smoked fish (60.2 g/day) and smoked‐dried fish (18.2 g/day) contributed for 112% (281.1 mg) and 72% (180.4 mg), respectively, to the adult daily recommended intake of sum of eicosapentaenoic acid (EPA) and docosahexaenoic acid (DHA) (250 mg/day). The daily protein intake related to the consumption of smoked fish and smoked‐dried fish corresponded to 36% and 24%, respectively, of the recommended intake suggested by European Food Safety Authority (0.8 g/kg.bw/day).

## Graphical abstract

Smoked and smoked‐dried fish are good sources of protein, lipid and semi‐essential fatty acids. The median consumption of smoked fish (60.2 g/day) and smoked‐dried fish (18.2 g/day) contributed for 72% and 112% respectively, to the adult daily requirement of sum of eicosapentaenoic acid (EPA) and docosahexaenoic acid (DHA) (250 mg/day).

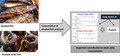

## 1 INTRODUCTION

Fishery products contribute to food security either by providing nutrients (protein, lipids, polyunsaturated fatty acids, vitamins, iodine, iron, etc.) to human body and by guaranteeing source of income for fishermen, processors, and countries (FAO (Food and Agriculture Organization of the United Nations), 2006, FAO (Food and Agriculture Organization of the United Nations), 2016a). The importance of fish and by‐products is shown by an increasing annual world‐wild fish consumption from 9.9 kg per capita to 19.7 kg per capita between 1960 and 2013 (FAO (Food and Agriculture Organization of the United Nations), 2016a, FAO (Food and Agriculture Organization of the United Nations), 2016b). In Benin, the average yearly consumption of fish per capita reached 15.9 kg in 2012 (El Ayoubi & Failler, 2013). Fish contributes for 17% to animal protein intake of world‐wide population and for 6.6% of total protein consumed (FAO (Food and Agriculture Organization of the United Nations), 2016b). In Benin, halieutic captures increased from 31,497 to 47,572 tons from 2005 to 2014. During the same period, importations of frozen fish increased from 45,228 to 163,126 tons. (FAO (Food and Agriculture Organization of the United Nations), 2016b; DP/MAEP, 2014). Among fish preservation methods, smoking is one of the most used (Alhassan, Boateng, & Ndaigo, 2012; Chabi et al., 2014; Kpodekon et al., 2014; Kumolu‐Johnson, Aladetohun, & Ndimele, 2010; Yakubu & Ngueku, 2015). According to Berkel, Boogaard, and Heijnen (2005), smoking is carried out according to three methods which are cold smoking (30°C), hot smoking (65–100°C) resulting in cooked but not dried product, and smoke drying at 45–85°C resulting in hot smoking followed by drying. In Benin, traditional hot smoking and smoke drying are the smoking methods frequently performed by female processors and used to preserve fish (Assogba et al., 2019). Smoked fish products are consumed all over the world as protein source (Alhassan et al., 2012; Desiere, Hung, Verbeke, & D'Haese, 2018; Kiczorowska, Samolinska, Grela, & Bik‐Małodzinska, 2019). Smoked fish has good nutritional quality including protein, lipid, and fatty acids (Kiczorowska et al., 2019; Nunoo, Tornyeviadzi, Asamoah, & Addo, 2019; Tiwo, Tchoumbougnang, Nganou, Kumar, & Nayak, 2019). Adeyeye, Fayemi, and Adebayo‐Oyetoro, (2018) and Amoussou et al. (2019) reported amino acids, vitamins, and oligoelements in raw and smoked fish. Fish products are also a good source of unsaturated fatty acids mainly the two essential fatty acids, namely alpha‐linolenic acid and linoleic acid (omega 3 and omega 6) and semi‐essential fatty acids, known as eicosapentaenoic acid (EPA) and docosahexaenoic acid (DHA) (Amoussou et al., 2019; Superior Health Council (SHC), 2016). Due to their nutritional quality, fish consumption results in important nutritional intake necessary for human health (Amoussou et al., 2019). Indeed, fish consumption is associated with reduction of death from coronary heart disease and reduction of colorectal cancer (Aglago et al., 2020; Clifton & Keogh, 2017). Although several studies were carried out on smoked fish and smoked‐dried fish (Adeyeye, Oyewole, Obadina, & Omemu, 2015; Amos & Paulina, 2017; Anihouvi et al., 2019; Assogba et al., 2019; Olayemi, Raji, & Adedayo, 2012), to our best knowledge, none of them provided relevant information on the quality of some of their nutrients, mostly the fatty acids, and their contribution to nutrients intake in the Beninese context. This study aims to assess the consumption of smoked fish and smoked‐dried fish produced in South Benin and to estimate the protein, lipid, and fatty acid daily intakes for adult in the study area.

## 2 MATERIAL AND METHODS

### 2.1 Study area, consumers, and data collection

Five municipalities of South Benin (Abomey‐Calavi, Aguégués, Aplahoué, Comè, and Cotonou) recognized as main localities in which fishery activities were intensively carried out, were chosen to conduct this study. Fifty (50) adult (≥18 years old) consumers were randomly selected per municipality and interviewed when they came for buying fish in market or on processing sites, based on the assumption that fish buyers are potential fish consumers. The consumer survey was conducted as individual face to face interview using semistructured questionnaire administrated in French or Benin local languages (*Goun*, *Fon*, *Mina*, and *Adja*). The questionnaire was composed of two sections. The first section was composed of questions related to name, age, body weight, gender, sociocultural group, educational level, religion, household size, and marital status. The second section was composed of questions related to the meal prepared out of smoked fish and smoked‐dried fish, the methods of cooking fish as consumed, other grilled or smoked products consumed, frequency and quantity, number of people who will eat the purchased fish, time of the day when smoked fish and smoked‐dried fish were consumed (breakfast, lunch, and dinner), places where smoked fish and smoked‐dried fish were consumed.

About fifteen (15) markets and twenty‐five fish smoking sites were visited during fifteen (15) days of survey. During this survey, the body weight of each interviewed consumer was taken using a balance (Seca LK N° 5,158). To calculate the quantity of smoked fish and smoked‐dried fish consumed by each consumer, questions were asked about the cost of smoked fish or smoked‐dried fish purchased and the number of people eating the quantity purchased. Similar quantity of smoked fish and smoked‐dried fish at the same cost was then purchased and weighed using calibrated balance and this value was used to estimate the individual amount of daily consumed smoked fish or smoked‐dried fish on the basis of the quantity consumed per meal and the consumption frequency recorded from the interviews. The daily consumption of smoked or smoked‐dried fish was estimated by dividing the quantity of fish purchased for daily consumption by the number of people at the household level.

### 2.2 Sampling and physico‐chemical analysis

Thirty‐six (36) samples of processed fish from three main fish species (*Scomber scombrus*, *Merluccius polli*, and *Oreochromis niloticus*) used to produce smoked fish and three main fish species (*Cypselurus cyanopterus*, *Sphyraena barracuda*, and *Ethmalosa fimbriata*) used to produce smoked‐dried fish were randomly sampled. For each species, six samples (03 on smoking sites and 03 on markets) were collected in the municipalities where consumption survey was conducted.

The moisture, protein, and lipid contents and the pH of collected samples were determined according to the methods described by Anihouvi, Ayernor, Hounhouigan, and Sakyi‐Dawson, (2006), ISO1442, 1973, Kjeldahl method (Norme Française, 2002) and Folch, Lees, and Sloane Stanley, (1957), respectively. The fatty acids were analyzed according to the method described by Douny et al. (2015) using a gas chromatography/mass spectrophotometry (GC/MS).

### 2.3 Daily intake estimation of macronutrients

One hundred and eighty‐six (186) consumers eating smoked fish or/and smoked‐dried fish with a minimum frequency of once per month were selected among the 250 interviewed fish consumers for the estimation of the protein, lipid, and fatty acids daily intakes. The average content of each macronutrient (expressed in % of wet weight) recorded for smoked fish and smoked‐dried fish was used to achieve this estimation. The calculated daily intake of protein (g/kg of body weight/day), lipid, and fatty acids (g/day) was then compared with a dietary reference value (DRV) recommended by international organizations (EFSA (European Food Safety Authority), 2010, 2012; FAO (Food and Agriculture Organization of the United Nations), 2010; Superior Health Council [SHC], 2016; World Health Organization (WHO), 2007).

### 2.4 Statistical analysis

Descriptive statistics were performed on the collected survey data using Sphinx survey plus2 (version 4.5) software and Microsoft Excel (2013) for laboratory data. The proportions of smoked fish and smoked‐dried fish consumers were compared using confidence intervals (CI) at 95%.

Kruskal–Wallis test was realized to show difference among the fish species composition, and significance was accepted at probability *p* < .05 using Statistica 7.1 (Statsoft France, 2006).

## 3 RESULTS AND DISCUSSION

### 3.1 Characteristics of consumers and factors associated with the consumption of smoked fish and smoked‐dried fish

Table 1 presents socio‐demographic characteristics of the interviewed consumers of smoked fish and smoked‐dried fish. The majority of the 250 surveyed consumers were aged ≤40 years (80.4%) and Christians (82.4 ± 4.7%). Most (74.4%) belonged to *Goun*, *Adja*, *Fon*, *Pedah*, and *Mina* sociocultural groups. They were from both genders with a higher proportion represented by women (61.2 ± 6.0%). The higher proportion of female consumers is explained by the fact that in Benin, women are the ones in charge of purchasing food from markets and mostly in charge of cooking food at household level. Most of the surveyed consumers (76%) had formal education comprising primary school (24.0%), secondary school (27.6%), and university level (24.4%). People with no formal education represented 24.0%. There was no significant (*p* > .05) difference between proportions of people of different educational levels. The marital status showed that most of them (55.2%) were married. The interviewed consumers claimed to consume fish under different processed forms such as smoked fish (96.8% of the respondents), fried fish (80.8%), smoked‐dried fish (68.4%), fresh fish cooked in sauce (58.4%), sun‐dried fish (28.0%), and roasted fish (0.4%) (Data not shown). These figures show the highest importance of smoked fish in the Beninese diet. Smoked fish and smoked‐dried fish are mainly consumed in cooked tomato or vegetable sauce (98.4%), followed by incorporation in uncooked or slightly cooked tomato sauce called *monyo* (79.6%), or in a fried‐tomato sauce called *Odja* (2%), (Data S1). Among smoked fish and smoked‐dried fish consumers, 26% claimed to consume fish with some crushed ingredients including tomato, onion, and pepper with sometimes addition of *afitin* (fermented African locust beans (*Parkia bigloboza)* used as condiment). Smoked fish and smoked‐dried fish are consumed with “*wô*,” a cooked maize dough (92.8% of consumers); diverse cooked fermented maize doughs like ‟*akassa*,” “*come*,” and “*lio”* (72.4%; 1.2% and 0.8% of consumers, respectively); cooked rice (53.6%); a cooked rice and bean product named “*atassi*,” (2.4%); cooked wheat products (11.6%) and pounded yam (3.6%) (Data S1). Many cassava‐based foods like “*agbéli*,” “*lafun*,” *gari*, and *klaklou* are also often consumed with smoked fish and smoked‐dried fish (Data S1).

**Table 1:** Socio‐demographic characteristics of the 250 surveyed consumers in the study area

Variables and modalities	Surveyed consumers number (*n* = 250)	Proportion (% ± CI)
Age (years)		
<20	34	13.6 ± 4.2^A^
20–30	114	45.6 ± 6.2^B^
31–40	53	21.2 ± 5.1^C^
41–50	31	12.4 ± 4.1^A^
51–60	13	5.2 ± 2.8^D^
>60	5	2 ± 1.7^E^
Gender		
Male	97	38.8 ± 6.0^A^
Female	153	61.2 ± 6.0^B^
Sociocultural groups		
Adja	56	22.4 ± 5.2^A^
Aïzo	6	2.4 ± 1.9^D^
Fon	33	13.2 ± 4.2^B^
Goun	58	23.2 ± 5.2^A^
Mina	18	7.2 ± 3.2^C^
Nago	9	3.6 ± 2.3^D^
Others^a^	17	6.8 ± 3.1^C^
Pédah	21	8.4 ± 3.4^C^
Sahouè	8	3.2 ± 2.2^D^
Sètô	6	2.4 ± 1.9^D^
Tori	9	3.6 ± 2.3^D^
Yoruba	9	3.6 ± 2.3^D^
Educational level		
Primary school	60	24.0 ± 5.3^A^
Secondary school	69	27.6 ± 5.5^A^
University	61	24.4 ± 5.3^A^
No school	60	24.0 ± 5.3^A^
Marital status		
Unmarried	110	44.0 ± 6.2^A^
Married	138	55.2 ± 6.2^B^
Divorcee	2	0.8 ± 1.1^C^
Religion		
Animism	31	12.4 ± 4.1^A^
Christianism	206	82.4 ± 4.7^B^
Islam	13	5.2 ± 2.8^C^

Note

Capital letters were used to point out significant difference among proportions of the same modality in different municipalities.

^a^ Others represent total of sociocultural characteristics which proportion is below 2%.

Smoked fish and smoked‐dried fish are consumed daily by 21.6% of surveyed consumers. The respondents claimed to consume smoked fish or smoked‐dried fish at home (100%), during lunchtime (89.2%) and during dinner (99.2%) (Table 2).

**Table 2:** Moment and place of smoked fish and smoked‐dried consumption fish (*n* = 250)

Consumption of smoked fish and smoked‐dried fish	Percentage of consumers (%)
Frequency of consumption	
Rarely	0.4
1−10/month	22.8
11−24/month	55.2
Daily	21.6
Moment of consumption	
Breakfast	47.2
Lunch	89.2
Dinner	99.2
Place of consumption	
Home	100
Open road side kiosk	30.8
Restaurants	6.8
Other places (market, school, work places, and ceremony places)	6

Most smoked fish and smoked‐dried fish consumers (98%) declared to consume other smoked or grilled products. They include grilled peanuts (78%), grilled maize (65.2%), roasted sheep meat “*tchachanga”* (60%), smoked cattle meat skin “*kpanman”* (56%), roasted chicken (76%), grilled pork (39.6%), grilled banana (24.8%), roasted cattle meat (16.4%), and roasted gizzard (8%) (Data S2).

### 3.2 Physico‐chemical characteristics of smoked fish and smoked‐dried fish

The physico‐chemical characteristics of the fish products are presented in Table 3. The moisture content recorded in the fish species studied varied between 18.9 ± 7.6% (*E. fimbriata*) and 67.2 ± 5.0% (*M. polli*) with the lowest moisture content recorded in the smoked‐dried fish species. The moisture content of *S. scombrus* is significantly higher (*p* < .05) than that of *C. cyanopterus* and *E. fimbriata* while the moisture content of *M. polli* is significantly higher than that of the three species of smoked‐dried fish. The lower moisture content recorded in smoked‐dried fishes when compared to the smoked fishes is due to the drying step which follows smoking process during smoked‐dried fish production, leading to an important water loss from the products. Regarding the moisture content of *E. fimbriata* (18.9 ± 7.6%), similar value was reported by Ojimelukwe, Ekong, and Akachukwu (2017) in smoked *E. fimbriata* (18.7 ± 0.02%). Idah and Nwankwo (2013) recorded an average moisture content of 45% in smoked *Oreochromis niloticus* which is not so far from the 53.5 ± 15.5% recorded in the present study for the same species. The Codex Alimentarius (Codex Stan 311–2013) recommended that moisture content of smoked‐dried fish should be ≤10% to guarantee microbial safety (absence of pathogenic bacteria and fungal spoilage) of the product (Alimentarius, 2013).

**Table 3:** Physico‐chemical characteristics of smoked fish and smoked‐dried fish according to fish species

Type of fish	Fish species (*n* = 6)	Moisture (%)	pH	% of dry weight	
				Protein	Lipid
Smoked fish	*Scomber scombrus*	63.6 ± 2.5^ac^	6.4 ± 0.3^ac^	59.0 ± 9.2^a^	39.0 ± 9.2^a^
	*Merluccius polli*	67.2 ± 5.0^a^	7.3 ± 0.6^b^	79.5 ± 6.6^ac^	19.4 ± 8.6^ac^
	*Oreochromis niloticus*	53.5 ± 15.5^abc^	7.2 ± 0.5^bd^	82.1 ± 2.7^ac^	17.4 ± 3.9^ac^
Smoked‐dried fish	*Cypselurus cyanopterus*	20.2 ± 6.1^bd^	6.5 ± 0.2^acd^	85.1 ± 2.3^bc^	11.0 ± 5.2^bc^
	*Sphyraena barracuda*	32.4 ± 12.5^cd^	6.8 ± 0.2^abc^	84.5 ± 4.2^bc^	14.4 ± 4.6^bc^
	*Ethmalosa fimbriata*	18.9 ± 7.6^bd^	6.7 ± 0.2^abc^	74.1 ± 6.7^ac^	22.1 ± 6.3^ac^

Note

Mean ± standard deviation; Means with different letters according to each column are significantly different (*p* < .05); *n*: number of samples analyzed per species.

The pH values recorded on the fish products ranged between 6 and 7. The pH value recorded on *M. polli* was significantly higher (*p* < .05) than the values recorded on *S. scombrus* and *C. cyanopterus*. Additionally, the pH value recorded on *O. niloticus* was also significantly higher (*p* < .05) than the value recorded on *S. scombrus*. This variability of pH between the fish products could depend on the species, but also on the postcapture preservation treatment. The pH value (6.4) recorded in smoked *S. scombrus* in this study is similar to the pH (6.1) reported by Kiczorowska et al. (2019) in the same specie smoked in Poland.

The average protein content of the six fish species is over 50% (dry matter) (Table 3). The protein content of smoked *S. scombrus* (59.0 ± 9.2%) in this study is similar to the one (63.7 ± 4.7%) recorded in the same species by Aremu, Namo, Oko, Adelagun, and Yebpella (2014), but significantly lower (*p* < .05) than the protein contents of *C. cyanopterus* and *S. barracuda*. The protein content recorded in smoked‐dried *S. barracuda* in this study (84.5%) is higher than the protein content of smoked *S. sphyraena* (70.8%), as reported by Nunoo et al. (2019).

The lipid content (% of dry matter) of the six fish species (Table 3) ranged between 11.0 ± 5.2% (for *C. cyanopterus*) and 39.0 ± 9.2% (for *S. scombrus*). The lipid content of *S. scombrus* was significantly higher (*p* < .05) than that of *C. cyanopterus* and *S. barracuda*. The lipid content of smoked *E. fimbriata* in this study (22.1%) is similar to the value reported by Ojimelukwe et al. (2017) in smoked *E. fimbriata* (20.2%).

The variability of the lipid content of the fish sample might be due to the fish species and the lipid loss during processing. Moreover, the use of vegetable oil by processors to make fish bright and attractive to buyers (Assogba et al., 2019) might also influence the lipid content of these fishes. No significant difference was recorded in the lipid and protein contents inside fish species undergoing the same treatment (smoked or smoked‐dried fish).

### 3.3 Fatty acids composition of smoked fish and smoked‐dried fish

The fatty acids composition varied widely among the 36 samples of smoked and smoked‐dried fish (Data S3). According to species, palmitic acid (C16:0) and stearic acid (C18:0) are the predominant saturated fatty acids (SFA), ranging between 25.0% and 41.3%, and between 5.0% and 13.4% of total fatty acids, respectively (Data S3). Among the monounsaturated fatty acids (MUFA), oleic acid (C18:1, w9) was the most abundant and ranged between 11.4% and 27.1% of total fatty acids. Furthermore, both omega 3 eicosapentaenoic acid (EPA) (C20:5) and docosahexaenoic acid (DHA) (C22:6) were the main contributors to the total polyunsaturated acids (PUFA) content (Data S3). Eicosapentaenoic acid (EPA; C20:5, omega 3) and docosahexaenoic acid (DHA; C22:6, omega 3) are two semi‐essential fatty acids derived from alpha‐linolenic acid. In this study, 66.6% of the samples (24 out of 36) had EPA between 1.1% and 12.5% of the total fatty acids while 94.4% of the samples (34 out of 36) had DHA between 0.6% and 33.2% of the total fatty acids (Data S3). Fish is recognized to be a good source of EPA and DHA, and for this reason, several authors used these two omega‐3 PUFA to appreciate the quality of fatty acids in fish (Kaya, Turan, & Emin erdem, 2008; Superior Health Council (SHC) 2016; Stołyhwo, Kołodziejska, & Sikorski, 2006). DHA and EPA consumption contributes together to reduce the risk of fatal ischemic heart disease (Lemaitre et al., 2003). Literature reported that the consumption of omega‐3 PUFA contributes to prevent age‐related macular degeneration (AMD) (Chong, Kreis, Wong, Simpson, & Guymer, 2008; Seddon, George, & Rosner, 2006). According to Albert et al. (2002), regular consumption of omega‐3 PUFA contained in fish reduces the risk of sudden death related to cardiovascular illness. Fish consumption is also associated with reduction of death from coronary heart disease and reduction of colorectal cancer (Aglago et al., 2020; Clifton & Keogh, 2017).

Specifically, the sum of the SFA in the smoked fishes (Table 4 and Data S3) ranged between 28.5% and 63.9% of the total fatty acids while the sum of the MUFA ranged between 17.8% and 43.4% of the total fatty acids and the sum of the PUFA between 4.2% and 53.6% of the total fatty acids. Regarding the smoked‐dried fishes (Table 4 and Data S3), the sum of the SFA ranged between 39.0% and 70.8% of the total fatty acids while the sum of the MUFA ranged between 12.6% and 39.0% of the total fatty acids and the sum of the PUFA between 2.8% and 46.5% of the total fatty acids.

**Table 4:** Fatty acids proportion (% of total fatty acids) of smoked fish and smoked‐dried fish according to fish species

Fatty acids	*Scomber scombrus*	*Merluccius polli*	*Oreochromis niloticus*	*Sphyraena barracuda*	*Ethmalosa fimbriata*	*Cypselurus* cyanopterus
SFA	56.5 ± 5.6	32.8 ± 4.4	53.9 ± 8.5	54.9 ± 10.1	60.7 ± 11.6	42.1 ± 3.2
MUFA	36.1 ± 4.4	27.9 ± 6.2	30.1 ± 4.6	28.9 ± 6.9	28.9 ± 4.3	14.8 ± 1.4
PUFA	7.4 ± 2.6	39.2 ± 10.0	16.0 ± 7.3	16.2 ± 14.6	10.5 ± 8.9	43.1 ± 3.8
Omega 6	2.3 ± 0.2	5.9 ± 1.6	7.8 ± 3.2	4.0 ± 2.2	4.5 ± 2.6	8.5 ± 0.8
Omega 3	5.1 ± 2.5	33.3 ± 11.2	8.2 ± 4.9	12.3 ± 12.6	8.9 ± 5.9	34.6 ± 3.4

Note

Mean value ± standard deviation.

Abbreviations: MUFA, Monounsaturated fatty acids; PUFA, Polyunsaturated fatty acids; SFA, Saturated fatty acids.

### 3.4 Macronutrients intake from smoked fish and smoked‐dried fish consumption

The consumption of smoked fish among the interviewed consumers ranged between 2.4 and 539.5 g/day/person with a median of 60.2 g/day/person. Regarding the smoked‐dried fish, its consumption ranged between 0.5 and 592.1 g/day with a median of 18.2 g/day. The protein, lipid, and fatty acids contents of individual samples of smoked fish and smoked‐dried fish used to estimate the daily intake of these nutrients are presented as Data S4 and Data S5, respectively. The daily intake of protein, lipid, SFA, MUFA, PUFA, omega 6, omega 3 and sum of EPA and DHA through the consumption of both smoked fish and smoked‐dried fish are summarized in Table 5.

**Table 5:** Macronutrients (Protein, lipid, and fatty acids) daily intake through consumption of smoked fish and smoked‐dried fish in Benin according to surveyed declarations

Parameters	Macronutrient daily intake per person from smoked fish and smoked‐dried fish consumption															
	Protein (g/kg bw/day)^a^		Lipid (g/day)^b^		SFA (g/day)^c^		MUFA (g/day)^d^		PUFA (g/day)^e^		Omega−6 fatty acids (g/day)^f^		Omega−3 fatty acids (g/day)^g^		EPA + DHA (mg/day)^h^	
	CSF	CSDF	CSF	CSDF	CSF	CSDF	CSF	CSDF	CSF	CSDF	CSF	CSDF	CSF	CSDF	CSF	CSDF
Minimum	0.01	0.005	0.2	0.1	0.1	0.03	0.1	0.01	0.02	0.01	0.01	0.002	0.02	0.01	11.2	4.7
Median	0.3	0.2	5.8	2.2	2.0	1.0	1.4	0.5	0.6	0.3	0.2	0.1	0.4	0.2	281.1	180.4
Percentile 75	0.5	0.5	9.2	4.2	3.2	1.9	2.2	0.9	1.0	0.6	0.3	0.2	0.7	0.4	449.8	338.8
Percentile 97.5	1.8	2.2	40.8	28.3	14.3	12.6	9.7	5.8	4.3	4.0	1.4	1.1	2.9	2.9	1 984.5	2 304.9
Maximum	3.1	6.6	51.7	72.0	18.1	32.1	12.4	14.8	5.4	10.1	1.8	2.8	3.7	7.4	2 518.6	5 867.1

Note

Abbreviations: CSDF, consumption of smoked‐dried fish; CSF, consumption of smoked fish.

^a^ Dietary reference value. (DRV) = 0.83 g/kg.bw/day for both genders (EFSA (European Food Safety Authority), 2012, Superior Health Council (SHC), (2016)) and DRV = 0.75 g/kg.bw/day (World Health Organization (WHO), 2007).

^b^ DRV => 55 < 83–97 g/day for men and >44 <66–77 g/day for women (Superior Health Council (SHC), (2016)).

^c^ DRV = <28 g/day for men and <22 g/day for women (Superior Health Council (SHC), (2016)).

^d^ DRV = 28–55 g/day for men and 22–44 g/day for women (Superior Health Council (SHC), (2016)).

^e^ DRV = 14–28 g/day for men and 11–22 g/day for women (Superior Health Council (SHC), (2016)).

^f^ DRV = 11–22 g/day for men and 8.8–18 g/day for women (Superior Health Council (SHC), (2016)).

^g^ DRV = 2.8–5.6 g/day for men and 2.2–4.4 g/day for women (Superior Health Council (SHC), (2016)).

^h^ DRV = 250–500 mg/day for both genders (SHC, 2016) and DRV = 250 mg/day for both genders (EFSA (European Food Safety Authority), 2010; FAO (Food and Agriculture Organization of the United Nations), 2010).

The daily protein intake related to smoked fish ranged between 0.01 g/kg.bw/day and 3.1 g/kg.bw/day with a median of 0.3 g/kg.bw/day while in the case of smoked‐dried fish, it ranged between 0.005 g/kg.bw/day and 6.6 g/kg.bw/day with a median of 0.2 g/kg.bw/day. The third quartile (Percentile 75) of the daily protein intake (0.5 g/kg.bw/day) related to the consumption of both smoked fish and smoked‐dried fish represents 67% of adult protein requirement (0.75 g/kg.bw/day) as recommended by WHO (World Health Organization), (2007). The 97.5th percentile and the maximum daily protein intake related to the consumption of smoked fish and smoked‐dried fish, exceeded 0.75 g/kg.bw/day adult protein requirement as recommended by World Health Organization (WHO), (2007) and 0.8 g/kg.bw/day adult protein requirement as recommended by EFSA (European Food Safety Authority), (2012).

The daily lipid intake ranged between 0.2 g/day and 51.7 g/day with a median of 5.8 g/day when considering the minimum and maximum daily consumption of smoked fish (2.4 g/day and 539.5 g/day). In the case of the smoked‐dried fish (0.5 g/day and 592.1 g/day), the daily lipid intake ranged between 0.1 and 72.0 g/day with a median of 2.2 g/day. The lipid requirement for adults should range between 55 and 83 g/day for men, and between 44 and 66 g/day for women, according to Superior Health Council (SHC), (2016).

The daily SFA intake through smoked fish consumption ranged between 0.1 g/day and 18.1 g/day while the daily SFA intake through smoked‐dried fish consumption ranged between 0.03 g/day and 32.1 g/day. These ranges of daily SFA intake agreed with the recommended threshold of <22 g/day (for women) and <28 g/day (for men) (Superior Health Council (SHC), (2016)).

The daily MUFA intake related to the consumption of smoked fish ranged between 0.1 g/day and 12.4 g/day while it ranged between 0.01 g/day and 14.8 g/day when consuming smoked‐dried fish. The daily MUFA intake for the maximum consumption of smoked fish (539.5 g/day) and the maximum consumption of smoked‐dried fish (592.1 g/day) contributed to about 50% of adult recommended intake of MUFA, which ranges between 28 and 55 g/day (for men) and between 22 and 44 g/day (for women) (Superior Health Council (SHC), (2016)).

The daily PUFA intake through smoked fish consumption ranged between 0.02 g/day and 5.4 g/day while the daily PUFA intake through smoked‐dried fish consumption ranged between 0.01 g/day and 10.1 g/day. According to Superior Health Council (SHC), (2016), adult PUFA recommended intake is 14–28 g/day for men and 11–22 g/day for women.

The daily omega‐6 fatty acids intake through the consumption of smoked fish ranged between 0.01 g/day and 1.8 g/day while the daily omega‐6 fatty acids intake through the consumption of smoked‐dried fish ranged between 0.002 g/day and 2.8 g/day. The highest omega‐6 fatty acids intakes through the consumption of smoked or smoked‐dried fish contribute to about 20% of adult recommended intake of omega‐6 fatty acids which are 11–22 g/day for men and 8.8–18 g/day for women (Superior Health Council (SHC) (2016)).

The daily omega‐3 fatty acids intake through the consumption of smoked fish ranged between 0.02 g/day and 3.7 g/day and the daily intake through smoked‐dried fish ranged between 0.01 g/day and 7.4 g/day while levels of 2.8–5.6 g/day are recommended for men and 2.2–4.4 g/day for women (Superior Health Council (SHC) (2016)).

The daily intakes of SFA, MUFA, PUFA, omega‐6, and omega‐3 fatty acids for minimum, median, P75, and P97.5 levels of fish consumption were higher through smoked fish consumption than through smoked‐dried fish consumption. Finally, the daily EPA + DHA intake through smoked fish consumption ranged between 11.2 mg/day and 2,518.6 mg/day with a median of 281.1 mg/day and between 4.7 mg/day and 5,867.1 mg/day with a median of 180.4 mg/day, through smoked‐dried fish consumption. The median consumption of fish contributed for 112.4% (for smoked fish) and 72.2% (for smoked‐dried fish), the adult daily EPA + DHA recommended intake (250 mg/day) as recommended by EFSA (European Food Safety Authority) (2010) and FAO (Food and Agriculture Organization of the United Nations) (2010).

## 4 CONCLUSION

Smoked and smoked‐dried fish are a part of the meal at breakfast, lunch or dinner during the day in the South Benin. The median consumption of smoked fish and smoked‐dried fish is estimated at 60.2 g/day and 18.2 g/day, respectively. These fish products are good sources of protein, lipid, and fatty acids. Palmitic acid (C16:0) and stearic acid (C18:0) are the predominant saturated fatty acids. EPA and DHA are the most abundant omega‐3 PUFA, while oleic acid is the most abundant MUFA. The median daily protein intake from smoked fish and smoked‐dried fish consumption represents about 26%–40% of adult protein requirement (0.75 g/kg.bw/day, according to the World Health Organization). The median daily lipid intake from the consumption of smoked fish (5.8 g/day) and smoked‐dried fish (2.2 g/day) contributes to less than 10% of adult lipid requirement as suggested by the Superior Health Council (Belgium). Among the fatty acids, the median daily intake of the sum of EPA and DHA from the consumption of smoked fish (281.1 mg/day) and smoked‐dried fish (180.4 mg/day) contributes to more than 70% of the sum of EPA and DHA recommended intake for adult as suggested by the European Food Safety Authority and the Food and Agriculture Organization.

## CONFLICT OF INTEREST

The authors have no conflicts of interest to declare.

## ETHICAL APPROVAL

This study does not involve any human or animal testing.

## INFORMED CONSENT

Written informed consent was obtained from all study participants.

## Supporting information

Data S1‐S5
