# Supplementary material for: Consumption and physico‐chemical characteristics of smoked and smoked‐dried fish commonly produced in South Benin and contribution to recommended nutrient intakes
Source: Food Sci Nutr. 2020 Jul 19;8(9):4822–30. doi: 10.1002/fsn3.1763 (PMC7500773; doi:10.1002/fsn3.1763)
Supplement: Supplementary file 1 — Data S1‐S5 [file FSN3-8-4822-s001.docx]

**Supplementary data 1.** Factors associated to the consumption of smoked fish and smoked-dried fish (n=250)

| Accompaniments, ingredients and sauce consumed with smoked fish and smoked-dried fish | Proportion of consumers (%) |
| --- | --- |
| Accompaniments | |
| *Wô*, a cooked maize dough | 92.8 |
| *Akassa*, a cooked fermented maize dough | 72.4 |
| *Come,* another cooked fermented maize dough | 1.2 |
| *Lio,* another cooked fermented maize dough | 0.8 |
| Rice | 53.6 |
| *Atassi*, a cooked rice and bean product | 2.4 |
| Cooked wheat products | 11.6 |
| Pounded yam | 3.6 |
| *Agbéli*, cooked fermented cassava dough | 3.2 |
| *Lafun,* another cooked fermented cassava dough | 0.8 |
| *Gari*, granules of roasted fermented cassava flour | 6.8 |
| *Èba,* cooked dough from gari | 20.8 |
| *Klaklou,* snack from cassava flour | 1.2 |
| Ingredients or sauce in which fish is incorporated | |
| Cooked tomato or vegetables sauce | 98.4 |
| *Monyo* (uncooked or slightly cooked tomato sauce) | 79.6 |
| *Odja,* fried-tomato sauce | 2 |
| Crushed tomato + onion + pepper (+ eventually *afitin*, fermented African locus beans (*Parkia bigloboza) used as condiment*) | 26 |

**Supplementary data 2.** Other grilled or smoked products consumed by consumers of smoked fish and smoked-dried fish (n=250)

| Other grilled or smoked products consumed | Proportion of consumers (%) |
| --- | --- |
| Grilled peanuts | 78 |
| Roasted chicken | 76 |
| Grilled maize | 65.2 |
| *Tchachanga* (roasted sheep meat) | 60 |
| *kpanman* (smoked cattle meat skin) | 56 |
| Grilled pork | 39.6 |
| Grilled banana | 24.8 |
| Roasted cattle meat | 16.4 |
| Roasted gizzard | 8 |
| Others* (cassava, sweet potatoes, rabbit, cat, dog, rat and greater cane rat) | 7 |

*****Others represent different grilled or smoked products which proportion is below 2%

**Supplementary data 3.** Fatty acids profile (% of total fatty acids) of individual samples of smoked fish (n°1 to 18) and smoked-dried fish (n°19 to 36). Samples n°1 to 12

| Group | Fatty acids | 1 | 2 | 3 | 4 | 5 | 6 | 7 | 8 | 9 | 10 | 11 | 12 |
| --- | --- | --- | --- | --- | --- | --- | --- | --- | --- | --- | --- | --- | --- |
| SFA | Capric acid (C10:0)  Lauric acid (C12:0) | <LOQ  <LOQ | <LOQ  <LOQ | <LOQ  <LOQ | <LOQ  <LOQ | <LOQ  <LOQ | <LOQ  <LOQ | <LOQ  <LOQ | <LOQ  <LOQ | <LOQ  <LOQ | <LOQ  <LOQ | <LOQ  <LOQ | <LOQ  <LOQ |
|  | Tridecylic acid (C13:0) | <LOQ | <LOQ | <LOQ | <LOQ | <LOQ | <LOQ | <LOQ | <LOQ | <LOQ | <LOQ | <LOQ | <LOQ |
|  | Myristic acid (C14:0) | 2.2 | 3.8 | 2.8 | 1.7 | 1.3 | 2.7 | 8.7 | 5.0 | 6.5 | 6.0 | 7.4 | 9.8 |
|  | Palmitic acid (C16:0) | 32.1 | 23.0 | 24.4 | 29.3 | 21.2 | 22.6 | 39.2 | 36.3 | 40.5 | 35.6 | 33.2 | 40.2 |
|  | Heptadecanoïc acid (C17:0) | <LOQ | <LOQ | <LOQ | <LOQ | <LOQ | 0.2 | 1.3 | 1.3 | 1.2 | <LOQ | 0.7 | 1.0 |
|  | Stearic acid (C18:0) | 4.3 | 4.2 | 5.0 | 6.8 | 6.0 | 3.4 | 11.9 | 13.4 | 10.0 | 11.7 | 6.2 | 10.7 |
|  | Arachidic acid (C20:0) | <LOQ | <LOQ | <LOQ | <LOQ | <LOQ | <LOQ | 1.4 | <LOQ | <LOQ | <LOQ | <LOQ | <LOQ |
|  | Docosanoïc acid (C22:0) | <LOQ | <LOQ | <LOQ | <LOQ | <LOQ | <LOQ | <LOQ | <LOQ | <LOQ | <LOQ | <LOQ | <LOQ |
|  | Lignoceric acid (C24:0) | <LOQ | <LOQ | <LOQ | <LOQ | <LOQ | <LOQ | <LOQ | <LOQ | <LOQ | <LOQ | <LOQ | <LOQ |
|  | **∑SFA** | **38.6** | **31.0** | **32.1** | **37.8** | **28.5** | **28.9** | **62.4** | **56.0** | **58.2** | **53.3** | **47.5** | **61.7** |
| MUFA | Palmitoleic acid (C16:1(omega 7)) | 2.7 | 5.0 | 4.6 | 3.3 | 2.2 | 6.2 | 6.3 | 7.7 | 8.3 | 7.4 | 4.4 | 12.0 |
|  | Heptadecenoïc acid (C17:1(omega 7)) | 0.6 | 1.0 | 1.0 | 0.9 | 0.7 | 1.0 | 1.2 | 1.3 | 1.4 | 1.4 | 0.9 | 2.0 |
|  | Oleic acid (C18:1(omega 9)) | 32.5 | 20.6 | 19.6 | 27.7 | 15.0 | 23.0 | 23.2 | 24.8 | 27.9 | 28.6 | 38.1 | 19.7 |
|  | **∑MUFA** | **35.8** | **26.6** | **25.2** | **31.9** | **17.9** | **30.2** | **30.7** | **33.8** | **37.6** | **37.4** | **43.4** | **33.8** |
| Omega 6_ PUFA | Linoleic acid (C18:2(omega 6)) | 6.7 | 2.1 | 2.6 | 4.7 | 2.3 | 2.2 | 2.2 | 2.2 | 2.1 | 2.3 | 1.6 | 2.4 |
|  | Gamma linolenic acid (C18:3(omega 6)) | <LOQ | <LOQ | <LOQ | <LOQ | <LOQ | <LOQ | <LOQ | <LOQ | <LOQ | <LOQ | <LOQ | <LOQ |
|  | Eicosadienoïc acid (C20:2(omega 6)) | <LOQ | 0.7 | <LOQ | <LOQ | <LOQ | <LOQ | <LOQ | <LOQ | <LOQ | <LOQ | <LOQ | <LOQ |
|  | Arachidonic acid (C20:4(omega 6)) | 1.5 | 2.5 | 2.2 | 2.8 | 3.6 | 1.8 | <LOQ | <LOQ | <LOQ | <LOQ | 1.0 | <LOQ |
| Omega 3_ PUFA | Alpha linolenic acid (C18:3(omega 3)) | <LOQ | 1.1 | 1.0 | <LOQ | <LOQ | 1.0 | <LOQ | <LOQ | <LOQ | <LOQ | 1.1 | <LOQ |
|  | Stearidonic acid (C18:4(omega 3)) | 1.5 | 1.7 | 1.7 | <LOQ | <LOQ | 1.9 | <LOQ | 1.4 | <LOQ | 2.0 | 1.6 | <LOQ |
|  | Di-homo-γ-linolenic acid (C20:3(omega 3)) | <LOQ | <LOQ | <LOQ | <LOQ | <LOQ | <LOQ | <LOQ | <LOQ | <LOQ | <LOQ | <LOQ | <LOQ |
|  | Eicosapentaenoïc acid (EPA.C20:5(omega 3)) | 6.3 | 10.9 | 11.4 | 5.3 | 11.5 | 12.5 | 2.2 | 2.2 | <LOQ | 2.5 | 1.2 | <LOQ |
|  | Docosapentaenoïc acid (DPA.C22:5(omega 3)) | 1.4 | 2.3 | 2.7 | 3.7 | 2.9 | 2.5 | <LOQ | 1.6 | <LOQ | <LOQ | 1.0 | <LOQ |
|  | Docosahexaenoïc acid (DHA.C22:6(omega 3)) | 8.2 | 21.1 | 21.2 | 13.8 | 33.2 | 18.9 | 2.5 | 2.9 | 2.1 | 2.4 | 1.7 | 2.2 |
|  | **∑PUFA** | **25.6** | **42.4** | **42.7** | **30.2** | **53.6** | **40.8** | **6.9** | **10.3** | **4.2** | **9.3** | **9.2** | **4.5** |

**SFA**: Saturated fatty acids; **MUFA**: Monounsaturated fatty acids; **PUFA**: Polyunsaturated fatty acids; **LOQ** (Limit of Quantification) = 0.1%; samples n° 1-6: *Merluccius polli*. 7-12: *Scomber scombrus*.

**Supplementary data 3.** (Contin.) Fatty acids profile (% of total fatty acids) of individual samples of smoked fish (n°1 to 18) and smoked-dried fish (n°19 to 36). Samples n°13 to 24

| Group | Fatty acids | 13 | 14 | 15 | 16 | 17 | 18 | 19 | 20 | 21 | 22 | 23 | 24 |
| --- | --- | --- | --- | --- | --- | --- | --- | --- | --- | --- | --- | --- | --- |
| SFA | Capric acid (C10:0) | <LOQ | <LOQ | <LOQ | <LOQ | <LOQ | <LOQ | <LOQ | <LOQ | <LOQ | <LOQ | <LOQ | <LOQ |
|  | Lauric acid (C12:0) | <LOQ | <LOQ | <LOQ | <LOQ | <LOQ | <LOQ | <LOQ | <LOQ | <LOQ | <LOQ | <LOQ | <LOQ |
|  | Tridecylic acid (C13:0) | <LOQ | <LOQ | <LOQ | <LOQ | <LOQ | <LOQ | <LOQ | <LOQ | <LOQ | <LOQ | <LOQ | <LOQ |
|  | Myristic acid (C14:0) | 4.4 | 2.8 | 2.6 | 6.4 | 3.1 | 1.4 | 1.6 | 2.1 | 2.5 | 0.8 | 2.4 | 1.5 |
|  | Palmitic acid (C16:0) | 32.3 | 37.0 | 32.6 | 47.5 | 40.2 | 37.1 | 25.3 | 23.6 | 30.6 | 24.1 | 22.2 | 24.2 |
|  | Heptadecanoïc acid (C17:0) | 1.3 | 1.1 | 0.7 | 0.9 | 2.1 | 0.7 | 1.6 | 2.0 | 1.9 | 1.1 | 1.9 | 1.7 |
|  | Stearic acid (C18:0) | 8.3 | 10.7 | 6.7 | 7.3 | 18.4 | 16.9 | 13.0 | 13.4 | 12.9 | 12.9 | 13.1 | 15.4 |
|  | Arachidic acid (C20:0) | <LOQ | <LOQ | 0.3 | 0.4 | <LOQ | <LOQ | <LOQ | <LOQ | <LOQ | <LOQ | 0.6 | <LOQ |
|  | Docosanoïc acid (C22:0) | <LOQ | <LOQ | <LOQ | <LOQ | <LOQ | <LOQ | <LOQ | <LOQ | <LOQ | <LOQ | <LOQ | <LOQ |
|  | Lignoceric acid (C24:0) | <LOQ | <LOQ | <LOQ | <LOQ | <LOQ | <LOQ | <LOQ | <LOQ | <LOQ | <LOQ | <LOQ | <LOQ |
|  | **∑SFA** | **46.3** | **51.7** | **42.9** | **62.4** | **63.9** | **56.1** | **41.4** | **41.1** | **47.9** | **39.0** | **40.1** | **42.9** |
| MUFA | Palmitoleic acid (C16:1(omega 7)) | 10.8 | 4.8 | 4.6 | 4.8 | 5.7 | 1.8 | 2.0 | 2.4 | 2.4 | 1.6 | 2.7 | 2.0 |
|  | Heptadecenoïc acid (C17:1(omega 7)) | 2.9 | 2.2 | 1.2 | 0.7 | 1.9 | 0.3 | 0.8 | 0.8 | 1.1 | 0.7 | 0.7 | 1.0 |
|  | Oleic acid (C18:1(omega 9)) | 16.7 | 23.8 | 26.4 | 25.0 | 13.8 | 33.0 | 9.8 | 11.3 | 12.6 | 12.2 | 13.1 | 11.7 |
|  | **∑MUFA** | **30.3** | **30.9** | **32.2** | **30.5** | **21.4** | **35.2** | **12.6** | **14.6** | **16.0** | **14.5** | **16.5** | **14.7** |
| Omega 6_ PUFA | Linoleic acid (C18:2(omega 6)) | 3.0 | 4.5 | 11.3 | 2.3 | 2.9 | 6.2 | 1.9 | 1.8 | 2.3 | 3.4 | 3.1 | 2.6 |
|  | Gamma linolenic acid (C18:3(omega 6)) | 1.2 | <LOQ | 0.6 | <LOQ | <LOQ | <LOQ | <LOQ | <LOQ | <LOQ | <LOQ | <LOQ | <LOQ |
|  | Eicosadienoïc acid (C20:2(omega 6)) | <LOQ | <LOQ | <LOQ | <LOQ | <LOQ | <LOQ | 0.8 | 0.9 | 1.1 | 0.7 | 0.7 | <LOQ |
|  | Arachidonic acid (C20:4(omega 6)) | 4.2 | 3.5 | 1.5 | 1.3 | 3.7 | 0.6 | 6.0 | 4.9 | 4.5 | 5.7 | 4.8 | 5.8 |
| Omega 3_ PUFA | Alpha linolenic acid (C18:3(omega 3)) | 1.8 | <LOQ | 1.0 | 0.9 | <LOQ | 0.9 | <LOQ | 0.8 | <LOQ | <LOQ | 1.3 | <LOQ |
|  | Stearidonic acid (C18:4(omega 3)) | 1.3 | <LOQ | 1.1 | 1.7 | <LOQ | 0.4 | <LOQ | <LOQ | <LOQ | <LOQ | <LOQ | <LOQ |
|  | Di-homo-γ-linolenic acid (C20:3(omega 3)) | <LOQ | <LOQ | <LOQ | <LOQ | <LOQ | <LOQ | <LOQ | <LOQ | <LOQ | <LOQ | <LOQ | <LOQ |
|  | Eicosapentaenoïc acid (EPA.C20:5(omega 3)) | 1.9 | <LOQ | 1.1 | <LOQ | <LOQ | <LOQ | 4.6 | 4.2 | 3.9 | 4.8 | 4.0 | 3.6 |
|  | Docosapentaenoïc acid (DPA.C22:5(omega 3)) | 6.1 | 4.1 | 5.2 | <LOQ | 3.9 | <LOQ | 3.8 | 6.2 | 4.7 | 3.5 | 5.6 | 4.2 |
|  | Docosahexaenoïc acid (DHA.C22:6(omega 3)) | 3.8 | 5.4 | 3.0 | 0.9 | 4.1 | 0.6 | 28.8 | 25.5 | 19.6 | 28.4 | 23.9 | 26.2 |
|  | **∑PUFA** | **23.4** | **17.4** | **24.9** | **7.1** | **14.7** | **8.7** | **46.0** | **44.3** | **36.0** | **46.5** | **43.3** | **42.4** |

**SFA**: Saturated fatty acids; **MUFA**: Monounsaturated fatty acids; **PUFA**: Polyunsaturated fatty acids; **LOQ** (Limit of Quantification) = 0.1%; samples n° 13-18: *Oreochromis niloticus*; 19-24: *Cypselurus cyanopterus*

**Supplementary data 3.** (Contin.) Fatty acids profile (% of total fatty acids) of individual samples of smoked fish (n°1 to 18) and smoked-dried fish (n°19 to 36). Samples n°25 to 36

| Group | Fatty acids | 25 | 26 | 27 | 28 | 29 | 30 | 31 | 32 | 33 | 34 | 35 | 36 |
| --- | --- | --- | --- | --- | --- | --- | --- | --- | --- | --- | --- | --- | --- |
| SFA | Capric acid (C10:0) | <LOQ | <LOQ | <LOQ | <LOQ | <LOQ | <LOQ | <LOQ | <LOQ | <LOQ | <LOQ | <LOQ | <LOQ |
|  | Lauric acid (C12:0) | <LOQ | <LOQ | <LOQ | <LOQ | <LOQ | <LOQ | <LOQ | <LOQ | <LOQ | <LOQ | <LOQ | <LOQ |
|  | Tridecylic acid (C13:0) | <LOQ | <LOQ | <LOQ | <LOQ | <LOQ | <LOQ | <LOQ | <LOQ | <LOQ | <LOQ | <LOQ | <LOQ |
|  | Myristic acid (C14:0) | 8.8 | 6.1 | 5.4 | 7.5 | 11.1 | 5.3 | 1.6 | 3.1 | 2.3 | 3.0 | 3.4 | 3.1 |
|  | Palmitic acid (C16:0) | 45.7 | 44.3 | 32.2 | 46.8 | 49.3 | 29.5 | 30.8 | 34.4 | 28.3 | 45.8 | 43.6 | 46.5 |
|  | Heptadecanoïc acid (C17:0) | <LOQ | 2.6 | 1.3 | 1.7 | <LOQ | 1.3 | 1.14 | 1.4 | 1.1 | 1.8 | 1.6 | 1.3 |
|  | Stearic acid (C18:0) | 9.1 | 14.9 | 8.3 | 10.4 | 10.3 | 8.3 | 12.3 | 12.5 | 8.5 | 13.8 | 12.1 | 12.2 |
|  | Arachidic acid (C20:0) | <LOQ | 0.7 | <LOQ | <LOQ | <LOQ | <LOQ | <LOQ | 0.5 | <LOQ | <LOQ | 0.5 | <LOQ |
|  | Docosanoïc acid (C22:0) | <LOQ | 0.7 | <LOQ | 0.6 | <LOQ | <LOQ | <LOQ | <LOQ | <LOQ | <LOQ | <LOQ | <LOQ |
|  | Lignoceric acid (C24:0) | <LOQ | 0.8 | <LOQ | 0.7 | <LOQ | 0.6 | <LOQ | 0.7 | 0.7 | 1.2 | <LOQ | <LOQ |
|  | **∑SFA** | 63.5 | 70.1 | 47.2 | 67.6 | 70.8 | 45.0 | 45.8 | 52.7 | 40.9 | 65.6 | 61.2 | 63.1 |
| MUFA | Palmitoleic acid (C16:1(omega 7)) | 17.2 | 9.6 | 18.0 | 10.4 | 17.0 | 18.1 | 2 | 3.1 | 4.7 | 4.5 | 3.8 | 6.0 |
|  | Heptadecenoïc acid (C17:1(omega 7)) | 2.2 | 1.4 | 3.5 | 1.8 | 2.4 | 3.3 | 0.9 | 0.7 | 0.9 | 1.0 | 0.7 | 1.1 |
|  | Oleic acid (C18:1(omega 9)) | 14.2 | 13.3 | 11.5 | 12.1 | 7.1 | 10.2 | 16.9 | 35.3 | 18.0 | 21.0 | 28.1 | 24.7 |
|  | **∑MUFA** | 33.5 | 24.4 | 33.0 | 24.3 | 26.4 | 31.6 | 19.8 | 39.0 | 23.6 | 26.5 | 32.6 | 31.8 |
| Omega 6_ PUFA | Linoleic acid (C18:2(omega 6)) | 3.0 | 1.9 | 3.2 | 2.6 | 2.8 | 3.3 | 3.4 | 2.3 | 1.6 | 1.1 | 1.4 | 1.4 |
|  | Gamma linolenic acid (C18:3(omega 6)) | <LOQ | <LOQ | 1.8 | <LOQ | <LOQ | 1.8 | <LOQ | <LOQ | 0.5 | <LOQ | <LOQ | <LOQ |
|  | Eicosadienoïc acid (C20:2(omega 6)) | <LOQ | <LOQ | <LOQ | <LOQ | <LOQ | <LOQ | <LOQ | <LOQ | <LOQ | <LOQ | <LOQ | <LOQ |
|  | Arachidonic acid (C20:4(omega 6)) | <LOQ | <LOQ | 2.8 | 1.3 | <LOQ | 2.8 | 4.3 | 1.4 | 3.3 | 1.8 | 1.3 | <LOQ |
| Omega 3_ PUFA | Alpha linolenic acid (C18:3(omega 3)) | <LOQ | 1.0 | 1.4 | 1.7 | <LOQ | 2.2 | <LOQ | <LOQ | <LOQ | <LOQ | <LOQ | <LOQ |
|  | Stearidonic acid (C18:4(omega 3)) | <LOQ | <LOQ | 1.4 | <LOQ | <LOQ | 1.8 | <LOQ | 2.3 | 0.9 | 1.3 | 1.5 | <LOQ |
|  | Di-homo-γ-linolenic acid (C20:3(omega 3)) | <LOQ | <LOQ | <LOQ | <LOQ | <LOQ | <LOQ | <LOQ | <LOQ | <LOQ | <LOQ | <LOQ | <LOQ |
|  | Eicosapentaenoïc acid (EPA.C20:5(omega 3)) | <LOQ | 1.1 | 3.7 | 1.1 | <LOQ | 5.2 | 3.7 | <LOQ | 4.9 | <LOQ | <LOQ | <LOQ |
|  | Docosapentaenoïc acid (DPA.C22:5(omega 3)) | <LOQ | <LOQ | 2.8 | <LOQ | <LOQ | 2.8 | 2.3 | <LOQ | 2.7 | <LOQ | <LOQ | 1.0 |
|  | Docosahexaenoïc acid (DHA.C22:6(omega 3)) | <LOQ | 1.6 | 2.8 | 1.3 | <LOQ | 3.6 | 20.7 | 2.4 | 21.5 | 3.6 | 2.0 | 2.7 |
|  | **∑PUFA** | 3.0 | 5.5 | 19.8 | 8.1 | 2.8 | 23.4 | 34.4 | 8.3 | 35.5 | 7.8 | 6.2 | 5.2 |

**SFA**: Saturated fatty acids; **MUFA**: Monounsaturated fatty acids; **PUFA**: Polyunsaturated fatty acids; **LOQ** (Limit of Quantification) = 0.1%; samples n° 25-30: *Ethmalosa fimbriata*; 31-36: *Sphyraena barracuda*

**Supplementary data 4.** Protein, lipid and fatty acid contents (g/100g wet weight) of individual samples of smoked fish (n=18), used to estimate the daily intake of the nutrients

| Samples | SFA | MUFA | PUFA | Omega 6 | Omega 3 | EPA+DHA | Lipid | Protein |
| --- | --- | --- | --- | --- | --- | --- | --- | --- |
| 1 | 2.2 | 2.0 | 1.4 | 0.5 | 1.0 | 0.8 | 11.9 | 27.2 |
| 2 | 1.0 | 0.9 | 1.4 | 0.2 | 1.2 | 1.0 | 4.2 | 34.3 |
| 3 | 0.8 | 0.6 | 1.0 | 0.1 | 0.9 | 0.8 | 4.4 | 24.9 |
| 4 | 0.9 | 0.8 | 0.7 | 0.2 | 0.6 | 0.5 | 7.9 | 20.2 |
| 5 | 0.4 | 0.2 | 0.7 | 0.1 | 0.6 | 0.6 | 3.6 | 26.1 |
| 6 | 1.2 | 1.3 | 1.7 | 0.2 | 1.5 | 1.3 | 6.2 | 24.0 |
| 7 | 3.9 | 1.9 | 0.4 | 0.1 | 0.3 | 0.3 | 16.0 | 19.6 |
| 8 | 2.5 | 1.5 | 0.5 | 0.1 | 0.4 | 0.2 | 10.9 | 22.9 |
| 9 | 8.1 | 5.2 | 0.6 | 0.3 | 0.3 | 0.3 | 15.1 | 21.9 |
| 10 | 5.0 | 3.5 | 0.9 | 0.2 | 0.7 | 0.5 | 13.4 | 21.1 |
| 11 | 9.2 | 8.4 | 1.8 | 0.5 | 1.3 | 0.6 | 21.1 | 17.9 |
| 12 | 7.9 | 4.3 | 0.6 | 0.3 | 0.3 | 0.3 | 9.7 | 24.8 |
| 13 | 2.6 | 1.7 | 1.3 | 0.5 | 0.8 | 0.3 | 5.9 | 31.4 |
| 14 | 2.4 | 1.5 | 0.8 | 0.4 | 0.4 | 0.3 | 5.4 | 42.9 |
| 15 | 5.6 | 4.2 | 3.3 | 1.8 | 1.5 | 0.5 | 14.4 | 63.2 |
| 16 | 3.2 | 1.6 | 0.4 | 0.2 | 0.2 | 0.04 | 6.7 | 26.5 |
| 17 | 1.9 | 0.7 | 0.4 | 0.2 | 0.2 | 0.1 | 8.7 | 32.7 |
| 18 | 1.7 | 1.1 | 0.3 | 0.2 | 0.1 | 0.02 | 7.2 | 33.0 |
| Minimum | **0.4** | **0.2** | **0.3** | **0.1** | **0.1** | **0.02** | **3.6** | **17.9** |
| Mean | **3.4** | **2.3** | **1.0** | **0.3** | **0.7** | **0.5** | **9.6** | **28.6** |
| Maximum | **9.2** | **8.4** | **3.3** | **1.8** | **1.5** | **1.3** | **21.1** | **63.2** |

**SFA:** Saturated fatty acids; **MUFA:** Monounsaturated fatty acids; **PUFA:** Polyunsaturated fatty acids; **LOQ** (Limit of Quantification) = 0.1%; samples n° 1-6: *Merluccius polli*, 7-12: *Scomber scombrus*, 13-18: *Oreochromis niloticus*

**Supplementary data 5.** Protein, lipid and fatty acid contents (g/100g wet weight) of individual samples of smoked-dried fish (n=18), used to estimate the daily intake of the nutrients

| Samples | SFA | MUFA | PUFA | Omega 6 | Omega 3 | EPA+DHA | Lipid | Protein |
| --- | --- | --- | --- | --- | --- | --- | --- | --- |
| 1 | 2.0 | 0.6 | 2.3 | 0.4 | 1.8 | 1.6 | 8.0 | 71.9 |
| 2 | 2.8 | 1.0 | 3.0 | 0.5 | 2.5 | 2.0 | 13.8 | 70.4 |
| 3 | 3.5 | 1.2 | 2.7 | 0.6 | 2.1 | 1.7 | 14.4 | 69.9 |
| 4 | 1.8 | 0.7 | 2.1 | 0.4 | 1.7 | 1.5 | 4.3 | 66.3 |
| 5 | 3.0 | 1.3 | 3.3 | 0.7 | 2.6 | 2.1 | 4.5 | 71.7 |
| 6 | 1.6 | 0.6 | 1.6 | 0.3 | 1.3 | 1.1 | 7.7 | 57.6 |
| 7 | 13.5 | 7.1 | 0.6 | 0.6 | <LOQ | <LOQ | 26.2 | 63.9 |
| 8 | 9.9 | 3.4 | 0.8 | 0.3 | 0.5 | 0.4 | 14.3 | 60.1 |
| 9 | 4.8 | 3.3 | 2.0 | 0.8 | 1.2 | 0.7 | 14.2 | 68.0 |
| 10 | 11.0 | 4.0 | 1.3 | 0.6 | 0.7 | 0.4 | 23.4 | 58.9 |
| 11 | 12.0 | 4.5 | 0.5 | 0.5 | <LOQ | <LOQ | 19.9 | 50.8 |
| 12 | 4.3 | 3.0 | 2.2 | 0.7 | 1.5 | 0.8 | 10.7 | 57.5 |
| 13 | 2.5 | 1.1 | 1.8 | 0.4 | 1.4 | 1.3 | 5.6 | 76.4 |
| 14 | 3.9 | 2.9 | 0.6 | 0.3 | 0.3 | 0.2 | 8.5 | 44.1 |
| 15 | 4.9 | 2.8 | 4.3 | 0.7 | 3.6 | 3.2 | 14.5 | 68.2 |
| 16 | 4.8 | 1.9 | 0.6 | 0.2 | 0.4 | 0.3 | 11.6 | 57.7 |
| 17 | 4.3 | 2.3 | 0.4 | 0.2 | 0.3 | 0.1 | 10.5 | 46.3 |
| 18 | 6.9 | 3.5 | 0.6 | 0.2 | 0.4 | 0.3 | 6.7 | 51.6 |
| Minimum | **1.6** | **0.6** | **0.4** | **0.2** | **0.3** | **0.1** | **4.3** | **44.1** |
| Mean | **5.4** | **2.5** | **1.7** | **0.5** | **1.4** | **1.1** | **12.2** | **61.7** |
| Maximum | **13.5** | **7.1** | **4.3** | **0.8** | **3.6** | **3.2** | **26.2** | **76.4** |

**SFA:** Saturated fatty acids; **MUFA:** Monounsaturated fatty acids; **PUFA:** Polyunsaturated fatty acids; **LOQ** (Limit of Quantification) = 0.1%; samples n° 1-6: *Cypselurus cyanopterus*, 7-12: *Ethmalosa fimbriata*, 13-18: *Sphyraena barracuda*
